# The human NMDA receptor GluN2A^N615K^ variant influences channel blocker potency

**DOI:** 10.1002/prp2.495

**Published:** 2019-06-20

**Authors:** Katie F. M. Marwick, Paul A. Skehel, Giles E. Hardingham, David J. A. Wyllie

**Affiliations:** ^1^ Centre for Discovery Brain Sciences Hugh Robson Building, University of Edinburgh Edinburgh UK; ^2^ Simons Initiative for the Developing Brain, Hugh Robson Building University of Edinburgh Edinburgh UK; ^3^ UK Dementia Research Institute University of Edinburgh Edinburgh UK; ^4^ Centre for Brain Development and Repair Institute for Stem Cell Biology and Regenerative Medicine Bangalore India

**Keywords:** blocker, channel, epilepsy, human, magnesium, mutation, NMDA, receptor, variant

## Abstract

*N*‐methyl‐D‐aspartate (NMDA) receptors are glutamate receptors with key roles in synaptic plasticity, due in part to their Mg^2+^ mediated voltage‐dependence. A large number of genetic variants affecting NMDA receptor subunits have been found in people with a range of neurodevelopmental disorders, including GluN2A^N615K^ (*GRIN2A* ^C1845A^) in two unrelated individuals with severe epileptic encephalopathy. This missense variant substitutes a lysine in place of an asparagine known to be important for blockade by Mg^2+^ and other small molecule channel blockers. We therefore measured the impact of GluN2A^N615K^ on a range of NMDA receptor channel blockers using two‐electrode voltage clamp recordings made in *Xenopus* oocytes. We found that GluN2A^N615K^ resulted in block by Mg^2+^ 1 mmol/L being greatly reduced (89% vs 8%), block by memantine 10 μmol/L (76% vs 27%) and amantadine 100 μmol/L (45% vs 17%) being substantially reduced, block by ketamine 10 μmol/L being modestly reduced (79% vs 73%) and block by dextromethorphan 10 μmol/L being enhanced (45% vs 55%). Coapplying Mg^2+^ with memantine or amantadine did not reduce the GluN2A^N615K^ block seen with either small molecule. In addition, we measured single–channel conductance of GluN2A^N615K^–containing NMDA receptors in outside‐out patches pulled from *Xenopus* oocytes, finding a 4‐fold reduction in conductance (58 vs 15 pS). In conclusion, the GluN2A^N615K^ variant is associated with substantial changes to important physiological and pharmacological properties of the NMDA receptor. Our findings are consistent with GluN2A^N615K^ having a disease–causing role, and inform potential therapeutic strategies.

**Abbreviations**
NMDA: *N*‐methyl‐D‐aspartate
WT: wild type

## 1 INTRODUCTION

*N*‐methyl‐D‐aspartate receptors are calcium‐permeable ligand‐gated glutamate receptors which unusually are blocked at rest by Mg^2+^ ions and thus also require postsynaptic depolarization in order to allow current flow.^1^, ^2^ This dual requirement for activation affords them crucial roles in synapse formation, plasticity and maintenance.^3^ Consistent with these important roles, NMDA receptor dysfunction has been linked to a range of neurodevelopmental and neurodegenerative disorders^3^ with a range of small molecules developed which influence NMDA receptor function and which are licensed for use in humans. The development of personalized medicine may allow these small molecules to be used in new situations, as clarifying the biological consequences of newly identified genetic variants allows repurposing of existing medications (for example 4).

NMDA receptors are heterotetramers containing two obligatory GluN1 subunits and two others, of which GluN2A and GluN2B are the commonest in the postnatal mammalian forebrain.^5^ Where two types of subunits are present the receptor is termed a diheteromer, where three types are present, a triheteromer. Over the last decade a large and increasing number of variants in the genes encoding NMDA receptor subunits have been identified in people with neurodevelopmental disorders including intellectual disability, schizophrenia, autism and epilepsy.^6^ Variants in *GRIN2A* are most likely to be associated with epilepsy aphasia syndromes, with the location and nature of the variant (loss of function vs missense) influencing the severity of the phenotype seen.^7^ The missense variant GluN2A^N615K^ is associated with a severe phenotype of early onset epileptic encephalopathy in two unrelated individuals.^8^, ^9^ It substitutes a lysine (positively charged) for an asparagine (neutral) in the M2 region of the NMDA receptor pore, in one of the narrowest regions of the pore.^10^ The residue affected is the most important determinant of Mg^2+^ block in GluN2A subunits: the “N + 1” site (an asparagine that neighbors the QRN site asparagine in GluN2A).^11^

Previous work has shown that the GluN2A^N615K^ variant has profound effect on NMDA receptor properties: it reduces block by Mg^2+^ 4, 9, 12 and influences block by other channel blockers,^4^ it reduces calcium permeability 9 and it reduces single–channel conductance.^12^ Importantly, the variant influences receptor properties even when only one copy is present in a receptor.^12^ Some of these effects could be viewed as “gain of function”, some “loss of function”. Seeking to reverse the “gain of function” component could be aided by the use of channel blockers, as has been trialed successfully by the use of memantine in a child carrying a different *GRIN2A* variant.^4^ To do this, detailed knowledge of the effect of channel blockers on receptors containing the GluN2A^N615K^ variant in physiological contexts is required.

In this study we therefore sought to replicate and extend previous work demonstrating a reduced potency of memantine and amantadine,^4^ by investigating the degree of inhibition by these blockers in the presence and absence of physiological concentrations of Mg^2+^. We examined the previously uninvestigated blocker ketamine. In addition, we replicated our previous finding of a reduction in single‐channel conductance^12^ in a different system using a different method of measurement. Our findings show that blocking GluN2A^N615K^ –containing NMDA receptors using memantine or amantadine remains possible in the presence of Mg^2+^, but that dextromethorphan is a more promising therapeutic candidate due to its increased inhibition in the presence of the variant.

## 2 METHODS

### 2.1 Test system used

*Xenopus laevis* oocytes were used in all experiments reported here. Experiments conducted during the course of this study received approval from the University of Edinburgh's Animal Welfare Ethical Review Board. Stage V‐VI oocytes were obtained from the UK Xenopus centre (Portsmouth,UK) and from Diaclean (CastropRauxel, Germany). Maintenance and culling of animals was performed by the oocyte providers. Approximately 200 oocytes were gathered from each of the eight *Xenopus laevis* used.

### 2.2 cRNA synthesis and expression in oocytes

The cDNA for wild type human NMDA subunit GluN1‐1a (hereafter GluN1) and GluN2A (GenBank accession codes: NP_015566, NP_000824)^13^ were gifts from Dr Hongjie Yuan (University of Emory). All cDNAs were in pCI‐neo. Site‐directed mutagenesis to generate GluN2A^N615K^ was performed as described previously^12^ using a mutagenizing polymerase chain reaction, recircularization and transformation. The mutation was verified using Sanger sequencing through the mutated region.

cRNA synthesis and expression was performed as described previously.^14^ cRNA for wild type and mutant subunits was synthesized from linearized plasmid DNA as runoff transcripts using the T7 polymerase mMessage mMachine RNA synthesis kit (Life Technologies Ltd, Paisley, UK). Each oocyte was injected with 3.7‐9 ng of cRNA, comprising a 1:1 molar ratio of GluN1 and GluN2A diluted in RNAse free water.

Prior to injection oocytes were collagenased (200 units/mL for 60 min), then manually defolliculated. After injection oocytes were placed in modified Barth's solution with composition (in mmol/L): 88 NaCl, 1 KCl, 2.4 NaHCO_3_, 0.82 MgCl_2_, 0.44 CaCl_2_, 0.33 Ca(NO_3_)_2_, 15 Tris‐HCl, adjusted to pH 7.35 with NaOH. This solution was supplemented with 50 IU/mL penicillin, 50 mg/mL streptomycin and 50 mg/mL tetracycline. Oocytes were then placed in an incubator (16‐21°C) for 24‐48 hours to encourage receptor expression and subsequently stored at 4°C.

Recordings were made 48‐96 hours post injection.

### 2.3 Measurements made

#### 2.3.1 Two‐electrode voltage‐clamp recordings

Two‐electrode voltage‐clamp recordings were performed as described previously.^14^ Recordings were made at room temperature (18‐21°C) from oocytes that were placed in a solution that contained (in mmol/L): 115 NaCl, 2.5 KCl, 10 HEPES, 1.8 BaCl_2_, 0.01 EDTA; pH 7.35 with NaOH. Recordings were made using a GeneClamp 500B amplifier (Molecular Devices, Union City, CA). Current and voltage electrodes were made from thin‐walled borosilicate glass (GC150TF‐7.5, Harvard Apparatus, Kent, UK) using a PP‐830 electrode puller (Narashige Instruments, Japan). Filling with 3 M KCl gave resistances of between 0.2 and 1.5 mol/L. Bath application of solutions was performed manually. Data were filtered at 10 Hz and digitized at 100 Hz via a 1401 plus analogue‐digital interface (Cambridge Electronic Design, Cambridge, UK) using WinEDR software (Strathclyde Electrophysiology Software, Strathclyde University, UK). Recordings were rejected if the holding current at –60 mV was greater than 150 nA or if the holding current drifted by more than 5% of the agonist response across the course of the experiment.

#### 2.3.2 Single‐channel voltage‐clamp recordings

Single‐channel voltage‐clamp recordings were made as described previously.^14^ Recordings were made at room temperature (18‐21°C) from outside‐out patches pulled from oocytes that were placed in a solution that contained (in mmol/L): 125 NaCl, 3 KCl, 1.25 NaH_2_PO_4_, 20 HEPES, 0.85 CaCl_2_, 0.01 EDTA; pH 7.35 with NaOH. Recording durations varied from 30 seconds to 5 minutes. Prior to recording vitelline membranes were removed from oocytes placed in a hypertonic solution that contained (in mmol/L): 200 sodium methyl sulfate, 20 KCl, 10 HEPES, 1 MgCl_2_, pH 7.4 with NaOH. Recordings were made using an AxoPatch 1D amplifier (Molecular Devices). Patch pipettes were made using thick walled borosilicate glass (GC150F‐7.5, Harvard Apparatus) using a P‐87 electrode puller (Sutter Instrument, Novato, CA) and their tips fire polished to give resistances of 7 to 12 MΩ when filled with internal solution containing (in mmol/L): 2.5 NaCl, 141 K‐gluconate, 10 HEPES, 11 EGTA; pH 7.4 with KOH. Electrode tips were coated in silicone elastomer (“Sylgard 184”, Dow Corning, Wiesbaden, Germany) to reduce capacitance. Application of solutions was controlled manually. Data were prefiltered at 2 kHz (–3 dB, 8th order Bessel filter) and digitized at 20 kHz via a Micro 1401 analogue‐digital interface (Cambridge Electronic Design) using WinEDR software (Strathclyde Electrophysiology Software). Patches were voltage‐clamped at –60 or –100 mV.

### 2.4 Data analysis and statistical procedures

WinEDR v3.3.7 (http://spider.science.strath.ac.uk/sipbs/software_ses.htm) was used to analyze all data. For two‐electrode voltage clamp recordings, steady state averages were taken for baseline, response to glutamate, and response to channel blockers, and percentage inhibition of the agonist response calculated. For single‐channel recordings, traces were idealized (using a transition threshold of 50% of the unitary conductance level and a 100 μs open and shut resolution) and amplitude histograms plotted. Gaussian curves were fitted to the currents seen in channel closed and open states, and the amplitude difference calculated. Conductance was calculated by dividing the current amplitude of an open channel by the holding potential. Any openings where more than one channel was open simultaneously were discarded.

Bar graphs depict individual cells (circles), means (columns) and standard error of the mean (error bars). R (v 3.1.2) (R Core Team, 2014) was used to perform statistical tests. Comparisons between multiple means were performed using ANOVA, with post hoc tests performed if the *F* test was significant for a main effect. Comparisons between two means were performed using independent, two‐tailed, Welch *t*‐tests (which do not assume equal variance between groups) unless otherwise stated. Correction for multiple comparisons were made using the Bonferroni method. The significance level used was *P* < 0.05. In figures, * indicates *P* < 0.05, ** indicates *P* < 0.01 and *** indicates *P* < 0.001.

### 2.5 Compliance with design and statistical analysis requirements

Oocytes were randomly allocated for injection with either WT or mutant cDNA. The experimenter was not blinded to construct as this was thought unlikely to influence the electrophysiological recordings. All n refer to number of oocytes, not number of traces. Although groups were designed to have equal n values, application of exclusion rules following recording (see above) meant that groups ended up unequal in size. The *t*‐tests used for comparisons adjust for resulting unequal variance. In the case of single‐channel recordings the n numbers reported are less than 5 but data obtained are highly consistent.

### 2.6 Drugs, reagents and other materials

Memantine, amantadine, ketamine, and dextromethorphan were purchased from Tocris Bioscience (Bristol, UK). The remaining substances were purchased from Sigma‐Aldrich (St Louis, MO).

## 3 RESULTS

### 3.1 GluN2A^N615K ^alters the inhibition of a range of NMDA receptor channel blockers, including Mg^2+^

**Figure 1:**
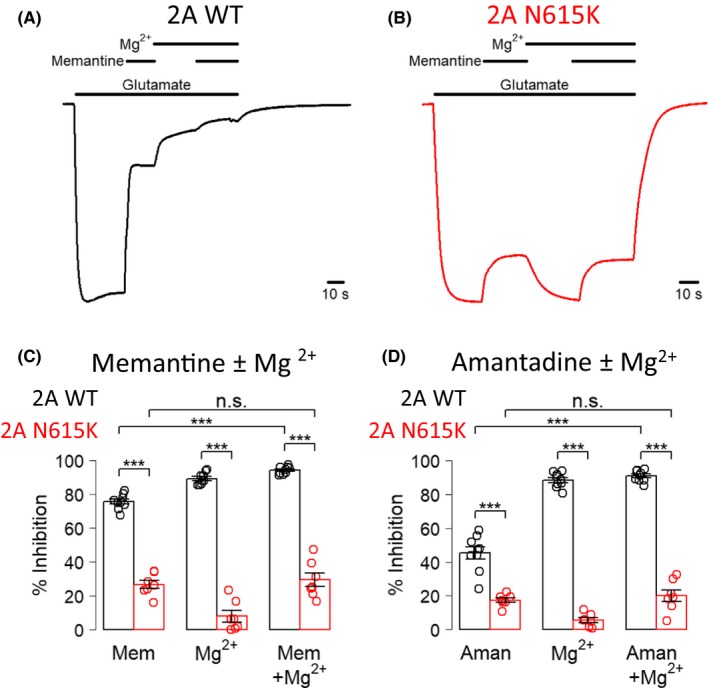
GluN2A^N615K^ reduces Mg^2+^, memantine and amantadine block. A and B, Representative two‐electrode voltage‐clamp recordings made from oocytes expressing GluN2A^WT^ or GluN2A^N615K^–containing NMDA receptors showing response to glutamate (30 μmol/L) and inhibition by memantine (10 μmol/L), Mg^2+^ (1 mmol/L) and the two combined, in the continuous presence of glycine (30 μmol/L). Holding potential –60 mV. C, Summary data showing percentage inhibition by memantine (10 μmol/L), Mg^2+^ (1 mmol/L) and the two combined for oocytes transfected with either GluN2A^WT^ or GluN2A^N615K^ –containing NMDA receptors, voltage‐clamped at −60 mV. A two‐way repeated measures ANOVA (blocker as within subjects factor, subunit as between subjects factor) showed a significant main effect of channel blocker (*F*_2,39_ = 26.1, *P* = 6.4E‐8) and of subunit (*F*_1,39_ = 588.3, *P* < 2E‐16) with a significant two‐way interaction (*F*_2,39_ = 7.3, *P* = 0.002). Planned post ‐hoc Bonferroni corrected independent Welch *t*‐tests showed that GluN2A^N615K^ was associated with lower blockade by memantine (WT: 76 ± 1%, N615K: 27 ± 2%, *t*_10.1_ = 17.1, *P* = 4.9E‐8), by Mg^2+ ^(WT: 89 ± 1%, N615K: 8 ± 3%, *t*_7.4_ = 23.2, *P* = 1.8E‐7) and by the two combined (WT: 94 ± 1%, N615K: 29 ± 4%, *t*_6.4_ = 15.7, *P* = 1.2E‐5). Planned post‐hoc Bonferroni corrected paired *t*‐tests showed that combining memantine and Mg^2+^ led to a higher degree of block in oocytes expressing GluN2A^WT^ subunits *t*_8_ = 17.5, *P* = 5.8E‐7) but not in those expressing GluN2A^N615K^ subunits (*t*_6_ = 1.4, *P* > 0.2) (WT: n = 9 oocytes, N615K: n = 7 oocytes). D, Summary data showing percentage inhibition by amantadine (100 μmol/L), Mg^2+^ (1 mmol/L) and the two combined for oocytes transfected with either GluN2A^WT^ or GluN2A^N615K^ –containing NMDA receptors, voltage‐clamped at –60 mV. A two‐way repeated measures ANOVA (blocker as within subjects factor, subunit as between subjects factor) showed a significant main effect of channel blocker (*F*_2,39_ = 146.5, *P* < 2E‐16) and of subunit (*F*_1,39_ = 481.8, *P* < 2E‐16) with a significant two‐way interaction (*F*_2,39_ = 20.0, *P* = 1E‐6). Planned post‐hoc Bonferroni corrected independent Welch *t*‐tests showed that GluN2A^N615K^ was associated with lower blockade by amantadine (WT: 45 ± 4%, N615K: 17 ± 1%, *t*_10.3_ = 7.3, *P* = 2.2E‐5), by Mg^2+ ^(WT: 88 ± 1%, N615K: 6 ± 1%, *t*_13.7_ = 39.9, *P* = 7.0E‐15) and by the two combined (WT: 91 ± 1%, N615K: 20 ± 4%, *t*_7.2_ = 19.2, *P* = 1.4E‐15). Planned post‐hoc Bonferroni corrected paired *t*‐tests showed that combining memantine and Mg^2+^ led to a higher degree of block in oocytes expressing GluN2A^WT^ subunits (*t*_8_ = 14.5, *P* = 2.5E‐6) but not in those expressing GluN2A^N615K^ subunits (*t*_6_ = 1.2, *P* > 0.3) (WT: n = 9 oocytes, N615K: n = 7 oocytes)

**Figure 2:**
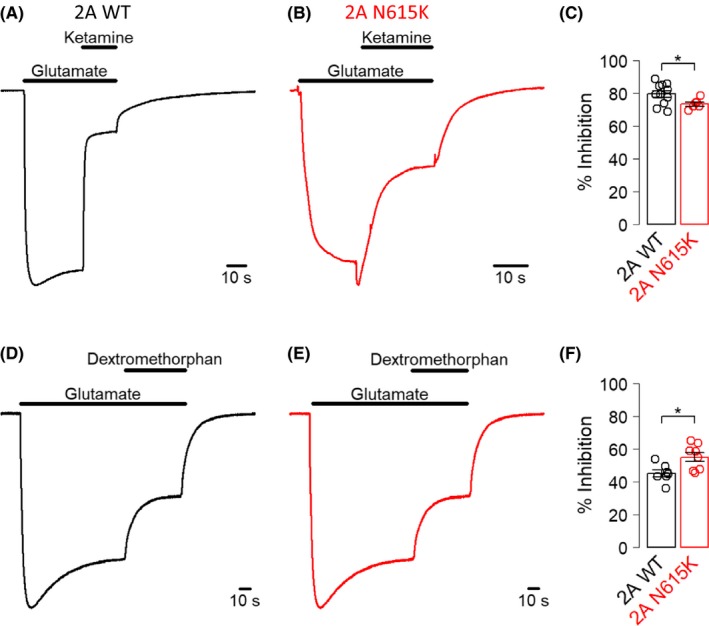
GluN2A^N615K^ reduces ketamine block but increases dextromethorphan block. A and B, Representative two‐electrode voltage‐clamp recordings made from oocytes expressing GluN2A^WT^ or GluN2A^N615K^–containing NMDA receptors showing response to glutamate (30 μmol/L) and inhibition by ketamine (10 μmol/L) in the continuous presence of glycine (30 μmol/L). Holding potential –60 mV. C, Summary data showing percentage inhibition by ketamine (10 μmol/L) for oocytes transfected with either GluN2A^WT^ or GluN2A^N615K^ –containing NMDA receptors, voltage‐clamped at –60 mV. An independent Welch *t*‐test showed that GluN2A^N615K^ was associated with a reduction in block (WT: 79 ± 2 (n = 11 oocytes), N615K: 73 ± 1 (n = 6 oocytes), *t*_14.9_ = 2.6, *P* = 0.019). D and E, Representative two‐electrode voltage‐clamp recordings made from oocytes transfected with GluN2A^WT^ or GluN2A^N615K^–containing NMDA receptors showing response to glutamate (30 μmol/L) and inhibition by dextromethorphan (10 μmol/L) in the continuous presence of glycine (30 μmol/L). Holding potential –60 mV. F, Summary data showing percentage inhibition by dextromethorphan (10 μmol/L) for oocytes transfected with either GluN2A^WT^ or GluN2A^N615K^ –containing NMDA receptors, voltage‐clamped at –60 mV. An independent Welch *t*‐test showed that GluN2A^N615K^ was associated with an increase in block (WT: 45 ± 2 (n = 7 oocytes), N615K: 55 ± 3 (n = 8 oocytes), *t*_12.6_ = 2.8, *P* = 0.015)

To assess the impact of the GluN2A^N615K^ mutation on inhibition by Mg^2+^ and small molecule channel blockers, we made two‐electrode voltage‐clamp recordings from oocytes expressing GluN2A^WT^ or GluN2A^N615K^ diheteromeric NMDA receptors exposed to glutamate (30 μmol/L) and glycine (30 μmol/L) while voltage‐clamped at –60 mV. We found that block by an approximately physiological concentration of Mg^2+^ (1 mmol/L) was markedly reduced in oocytes expressing GluN2A^N615K ^(Figure 1A‐D). We also found that the maximum percentage inhibition by saturating concentrations of memantine, amantadine and ketamine was reduced by the GluN2A^N615K^ variant (Figure 1A‐D, 2A‐C). Coapplying Mg^2+^ with memantine or amantadine did not reduce their ability to block GluN2A^N615K^–containing NMDA receptors (Figure 2C,D). In contrast to the other channel blockers, the maximal inhibition by dextromethorphan was modestly increased by the presence of the GluN2A^N615K^ variant (Figure 2D‐F).

### 3.2 GluN2A^N615K^ reduces single–channel conductance

**Figure 3:**
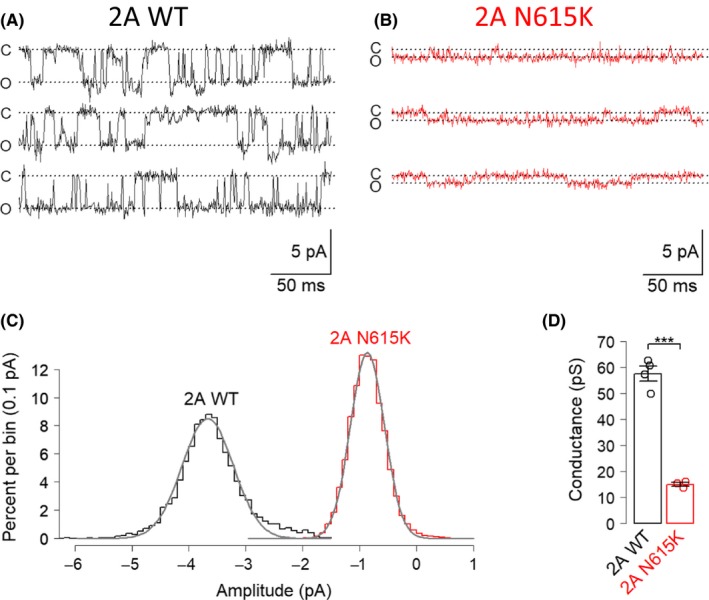
GluN2A^N615K^ reduces single‐channel conductance. A and B, Representative voltage‐clamp recordings made from outside‐out patches from oocytes expressing GluN2A^WT^ or GluN2A^N615K^ –containing NMDA receptors in the presence of glutamate (30 μmol/L) and glycine (30 μmol/L). “C” = closed, “O” = open. Holding potential −60 mV. C, Representative amplitude histograms showing fitted normal distributions, superimposed from two different patches held at –60 mV. The means of the fitted distributions/holding potential were used to calculate conductance. D, Summary data showing conductance, calculated as current amplitude/holding potential. A *t*‐test showed a reduction in conductance in oocytes expressing GluN2A^WT^ (58 ± 3 pS (n = 4), total events = 2555) and GluN2A^N615K^ (15 ± 1 pS (n = 3), total events = 774, *t*_3.4_ = 14.6, *P* = 0.0004)

In view of the profound influence of the GluN2A^N615K^ mutation on channel blockade we next assessed the variant's impact on ion permeation. In outside‐out patches pulled from oocytes expressing GluN2A^WT^ or GluN2A^N615K ^–containing NMDA receptors we identified a 4‐fold reduction in single–channel conductance (Figure 3).

## 4 DISCUSSION

### 4.1 GluN2A^N615K^ influences degree of blockade by NMDA receptor channel blockers

We found that GluN2A^N615K^ reduced the inhibition caused by the small molecule channel blockers memantine, amantadine and ketamine, while increasing the inhibition caused by dextromethorphan. This is consistent with previous work^4^ and with work identifying overlap between residues important for Mg^2+^ block and residues important for binding of memantine 10, 15 and ketamine.^16^ Amantadine is structurally very similar to memantine so is likely to interact with similar residues. In contrast, dextromethorphan's primary metabolite dextrorphan interacts with residues in the more extracellular portion of the vestibule.^17^ Our data suggest that this interaction is nonetheless altered by the GluN2A^N615K^ variant deeper in the receptor pore. This may be of potential therapeutic relevance as dextromethorphan is already licensed for use in humans (it is a common ingredient in cough medicines). However, the enhanced block of GluN2A^N615K^ –containing receptors by dextromethorphan would only be of benefit if the primary problem caused by the variant was receptor hyperfunction, rather than hypofunction. Our findings and previous work show that the variant is associated with alterations in receptor properties which could potentially lead to either outcome, so this would need to be investigated cautiously. Further, the increase in block in GluN2A^N615K^ –containing receptors is modest, meaning that the effects of any given dose of dextromethorphan on wild type receptors would also need to be considered and could potentially be harmful.

We also found that coapplying Mg^2+^ with the channel blockers memantine and amantadine had no effect on the maximal GluN2A^N615K^ block observed with the small molecules. This suggests that these drugs could still be expected to cause some block of GluN2A^N615K^ –containing NMDA receptors in vivo, but that they would cause more block at wild type receptors. This would likely limit their therapeutic utility, unless compensatory processes in some way caused wild type NMDA receptor overactivation. Our finding that wild type subunits show higher block when memantine or amantadine are coapplied with Mg^2+^ than when either are applied alone is consistent with work done in the same system with the same concentrations of blocker used.^18^

### 4.2 GluN2A^N615K^ reduces single–channel conductance

We found that GluN2A^N615K^ –containing NMDA receptors showed a 4‐fold reduction in single–channel conductance. The conductance value obtained here using technically “gold standard” outside‐out patches is in good agreement with that obtained using cell–attached patches containing GluN2A^N615K^ diheteromeric receptors expressed in human embryonic kidney (HEK293T) cells.^12^ The reduction in cation conductance seen likely reflects the introduction of a positively charged residue at a constriction of the pore.^10^ This region has already been shown to determine conductance by mutation studies in GluN1 and GluN2B subunits.^19^, ^20^, ^21^ The reduction in conductance is likely to disrupt the receptor's ionotropic signaling function and thus potentially impair both neurotransmission and plasticity. However, a reduction in conductance is unlikely to be amenable to therapeutic modification by existing small molecules.

In conclusion, this study found that the human disease–associated variant GluN2A^N615K^ has marked effects on inhibition by channel blockers and single–channel conductance. The channel blocker dextromethorphan is a potential candidate for possible therapeutic use.
